# Physiology limits commercially viable photoautotrophic production of microalgal biofuels

**DOI:** 10.1007/s10811-017-1214-3

**Published:** 2017-07-13

**Authors:** Philip Kenny, Kevin J. Flynn

**Affiliations:** Biosciences, Wallace Building, Swansea University, Singleton Park, Swansea, SA2 8PP UK

**Keywords:** Microalgae, Biomass, Biofuels, Modelling, Sustainability, Energy

## Abstract

Algal biofuels have been offered as an alternative to fossil fuels, based on claims that microalgae can provide a highly productive source of compounds as feedstocks for sustainable transport fuels. Life cycle analyses identify algal productivity as a critical factor affecting commercial and environmental viability. Here, we use mechanistic modelling of the biological processes driving microalgal growth to explore optimal production scenarios in an industrial setting, enabling us to quantify limits to algal biofuels potential. We demonstrate how physiological and operational trade-offs combine to restrict the potential for solar-powered algal-biodiesel production in open ponds to a ceiling of ca. 8000 L ha^−1^ year^−1^. For industrial-scale operations, practical considerations limit production to ca. 6000 L ha^−1^ year^−1^. According to published economic models and life cycle analyses, such production rates cannot support long-term viable commercialisation of solar-powered cultivation of natural microalgae strains exclusively as feedstock for biofuels. The commercial viability of microalgal biofuels depends critically upon limitations in microalgal physiology (primarily in rates of C-fixation); we discuss the scope for addressing this bottleneck concluding that even deployment of genetically modified microalgae with radically enhanced characteristics would leave a very significant logistical if not financial burden.

**Electronic supplementary material:**

The online version of this article (doi:10.1007/s10811-017-1214-3) contains supplementary material, which is available to authorized users.

## Introduction

The transport sector is expected to remain largely reliant on carbon-based energy systems for the foreseeable future (Caspeta et al. 2013) and hence needs to exploit energy sources derived from biomass if it is approach C-neutrality. Biofuel derived from terrestrial crops offers one solution but their production adds pressures on land demand and threatens food security (Runge and Senauer 2007; Timilsina and Shrestha 2011). The potential that algal biofuels presents has generated much enthusiasm and less controversy; algae do not directly compete with food crops as a product (microalgae-derived food remains of minor, niche, interest) while the potential for high production rates exploiting non-agricultural land also minimises conflicts with food security (Clarens et al. 2010). Furthermore, many of the carbon-rich cellular compounds microalgae accumulate under nitrogen-deplete conditions (carbohydrate and/or lipid) appear ideal for conversion to biodiesel and bioethanol (Schenk et al. 2008; Sing et al. 2013).

Despite much investigation, the real potential of algae biofuels remains uncertain (Sun et al. 2011; Stephens et al. 2013). This is due, in large part, to highly diverse productivity claims, ranging from a few thousand (Walker 2009; Ramachandra et al. 2013) to hundreds of thousands of litres of biodiesel per hectare per year (Chisti 2007; Mata et al. 2010). There are also significant discrepancies between the high rates claimed through modelling efforts versus lower production rates achieved in reality (Moody et al. 2014). In large measure, such discrepancies may reflect the typically over-simplistic modelling description of microalgal physiology deployed in theoretical studies (Kenny and Flynn 2016). While a matter of concern purely for biomass projections, such simplifications are of far greater concern for the modelling of biofuels production; this is because the accumulation of surplus C within cells that may be exploited for biofuels feedstocks occurs mainly during the growth phase when the supply of nitrogen (N) limits cell proliferation (Rodolfi et al. 2009). In consequence, maximum cellular growth rates (which typically correlate broadly in a linear fashion with cellular N/C; Flynn 2008) and the maximum content of biofuels precursors within cells are mutually exclusive as a function of microalgal cell physiology. To accumulate C-rich metabolites rapidly also requires cells to be well illuminated, which at the high biomass densities typical of commercial systems (required to minimise space and energy needs), requires optically thin (i.e. shallow) suspensions to minimise self-shading within the algal cell population. Unfortunately, optimisations for high areal and volumetric productivities also indicate that the optimal depth of cultivation systems for biofuels production is somewhat shallower than the minimum raceway depth required to enable a well-mixed, stable culture system (Tredici 2007; Ritchie and Larkum 2012; Kenny and Flynn 2015).

A key factor in modelling microalgal growth is the selection of parameter values constraining key physiological processes. The most important of these is that defining the biochemical limitation of the rate of C-fixation. This process is mediated by the enzyme ribulose-1,5-bisphosphate carboxylase/oxygenase (RuBisCO). RuBisCO has a rather low specific catalytic rate, a competing affinity for the by-product of photosynthesis (O_2_) and a need for CO_2_ as the substrate (thus requiring a carbon concentrating mechanism to optimise C-fixation). The analysis of Flynn and Raven (2017) indicates an upper plausible rate of sustained gross C-fixation under continuous illumination equivalent to a maximum C-specific growth rate (net C-fixation; *U*_m_) of the order of a few divisions per day. Such growth rate potentials contrast greatly with assumed maximum rates used in some models of microalgal productivity (e.g. De-Luca et al. 2016). It is also noteworthy that short-term C-fixation rates, and rates derived from analysis of the performance of the light-reactions of photosynthesis, have potential to far overestimate long-term (i.e. day-duration) rates of production (Flynn and Raven 2017). Worse still, under natural light, this biologically expensive and inefficient enzyme (Tcherkez et al. 2006) lays dormant for half the day (i.e. at night), and hence the C-specific growth rate attainable by typical microalgae in naturally lit waters is more likely ca. 1 day^−1^ (Flynn and Raven 2017).

Perhaps unsurprisingly, sensitivities within life cycle analyses (LCAs) attempting to quantify environmental impacts and commercial potential (Stephenson et al. 2010; Williams and Laurens 2010; Handler et al. 2012) have been dominated by uncertainties in the cost analysis attributed to algal lipid content and growth rates (Davis et al. 2011; Liu et al. 2012; Sills et al. 2013). In consequence, investigators often explore baseline/best-case scenarios for comparison. For instance, Sun et al. (2011) consider several scenarios assuming areal production of biofuels feedstocks (in the form of lipids) spanning the equivalent of 4 to 29 g biofuel-C m^−2^ day^−1^ [assuming an elemental C content of 0.75–0.8 gC (g lipid)^−1^; Williams and Laurens 2010; Bellou et al. 2014]. Only at the upper limit of this productivity range did they find that algal biofuels could even approach becoming commercially competitive with fossil fuels. These authors (Sun et al. 2011) concluded (as have others—Davis et al. 2011; Sills et al. 2013) that further technological and biological developments are needed to achieve viability. This current work develops this important debate by investigating whether a level of microalgal biofuel productivity approaching the viability threshold of ca. 30 g biofuel-C m^−2^ day^−1^ (Sun et al. 2011; Sills et al. 2013), equivelent to ca. 130,000 L biodiesel ha^−1^ year^−1^, is likely to be attainable assuming that all engineering and allied logistic limiting factors are optimised, or whether there are more fundamental biological barriers to us ever achieving such productivities on a commercial scale.

Here, we present a detailed exploration of the conflicting factors affecting commercial microalgal biofuels production at the level of algal physiology, and probe the limits that these impose on the viability and sustainability of this technology. To do so, we employed a mechanistic description of the biological processes driving microalgal growth operated under a range of conditions and harvesting regimes at an industrial scale that simulate solar-powered cultivation of natural strains in open ponds. While high standing-stock biomass is a function of nutrient loading, high productivity of the feedstock for biofuels requires a balance of irradiance and nutrient supply to the individual cells in the population. The challenge for algal biofuels production is simultaneously achieving high biomass (which risks self-shading and hence light-limiting photosynthesis) and high, yet N-stressed, productivity; the latter is required to stimulate high lipid content. Importantly, a failure to take into account dynamic effects of photoacclimation within a growing algal population (that collectively self-shades community photosynthesis), and nutrient uptake regulation, leads to an over-estimation of productivity. That likelihood is greater again if implausible growth rates are assumed in models (i.e. rates that exceed those supportable by potential RuBisCO activity).

Our model describes the physiological interactions at the heart of this complex process. The basis of the microalgal model is a well-established (Flynn 2008, 2010; Flynn et al. 2008) acclimative, mechanistic structure describing growth and changes in cellular stoichiometry (C/N/P/Chl) as driven by multi-nutrient and light availability (Flynn 2001). The content of carbohydrate and lipid available for support of biofuels generation is given by the model as the excess cellular-C content over that stoichiometrically bound to N for proteins (including RuBisCO, whose maximum activity is explicitly described in the model), DNA, RNA and allied membrane lipid. That biofuels potential is enhanced on N-limitation of growth. A demonstration of the full model (i.e. the physiology model coupled to a description of a culture system) operating against a published data set for long-term, pilot-scale biofuels feedstock production in a real system (Quinn et al. 2011) has been published (Kenny and Flynn 2016). Using this validated model, we have explored a broad range of scenarios, enabling the contrasting optimisation strategies required for biomass and biofuels production to be determined.

## Methods

### Model overview

The Electronic Supplemental Material contains full details of the model equations (Model_Info_equations), along with a schematic of the model structure (Model_Info_schematic) and a catalogue of its applications (Model_Info_deployment). The following briefly outlines some salient features.

In the model, we make the explicit assumption that growth conditions, other than light and nutrient-N availability, are optimal. Thus, we assume that pH is held optimal (most likely achieved in practice through the use of a CO_2_-stat that would simultaneously maintain non-limiting CO_2_ for photosynthesis and also flush out RuBisCO-inhibitory O_2_), and that temperature is also optimal. Temperature optimality is especially problematic in reality as evaporation from ponds lowers temperatures, while elevated temperatures (enhanced in dense cultures by light absorbance) stimulates significant elevated metabolism in the short term (Eppley 1972; Béchet et al. 2014) but in the longer term kills, or (over months) forces an adaptation to the new conditions (Droop 1974); in consequence, any gains from growth at higher temperature will be short-lived (Flynn and Raven 2017). In reality, and of increasing concern at higher latitudes because of increased seasonal and climatic variation, a microalgal strain selected to grow and survive at peak summer temperatures would grow less well during winter days. Pragmatically, the solution is to deploy different strains of microalgae at different times of the year (akin to a farmer growing different seasonal crops); here we have assumed that the use of alternate microalgal crops matching seasonal changes provides a seamless continuity in production over the year. To explore a range of possibilities, we have run our simulations at different maximum growth rates with emphasis in our Results section assuming either a typical high maximum growth rate, or a value equating to the maximum plausible long-term sustainable value (Flynn and Raven 2017).

The surface irradiance driving photosynthesis depends upon geographic location, time of year and atmospheric conditions. In the model, latitude informs a solar cycle function which simulates diurnal and seasonal variations in available natural light. This function is multiplied by an insolation clearness index obtained from NASA’s Surface Meteorology and Solar Energy database (eosweb 2014). The value, adjusted and averaged over each month for every degree of latitude accounting for factors such as cloud cover and dust, ranges between 0.45 and 0.7. The model thus accounts explicitly for the light/dark day-length periodicity (Kenny and Flynn 2015, 2016), such that while summer production at high latitude may be relatively high, over the whole year production is markedly decreased by low winter irradiance due to shorter days, declination angle of the sun and typically also by increased cloud cover. We assumed that commercial facilities deploying large open ponds would maximise light availability by avoiding any obstructions to direct sunlight; hence, diffuse irradiance is neglected in this instance (see Kenny and Flynn 2016). Total daily photosynthetic activity is calculated by integrating over the optical depth which, assuming a shallow homogeneous cell suspension, we equate here to the pond depth, the diameter of photobioreactor tubes or gap between bioreactor plates. The light attenuation factor of the culture suspension is a function of pigment concentration, which itself depends upon the total biomass and the cellular Chl/C quota; these are affected, in turn, by the dynamics of the cells’ nutrient status. The carbon-specific algal growth rate dynamically balances photosynthetic gains versus respiratory losses, the latter being indexed to the maximum growth rate, *U*_m_, and the cells’ N/C status (Flynn 2001).

The core equations describing the microalgae submodel are as follows:

The carbon-specific growth rate is designated as *Cu*. For each nutrient, *X*_*i*_ (where *i* denotes N, P etc.), the quotient describing the relevant growth rate, *X*_*i*_ *Cu*, has the general form:

1 $$ {X}_i C u=\frac{\left(1+{KQX}_i\right)\left({X}_i C-{X}_i{C}_0\right)}{\left({X}_i C-{X}_i{C}_0\right)+{KQX}_i\left({X}_i{C}_m-{X}_i{C}_0\right)} $$

with *X*_*i*_ *C* denoting the C-quota for each nutrient while *X*_*i*_ *C*_0_ and *X*_*i*_ *C*_*m*_ are the quotas necessary to sustain minimum and maximum growth, respectively. *KQX*_*i*_ acts like a half-saturation constant affects the curve shape. At each timestep, this quotient is used to test (through Boolean logic terms) which, if any, of the nutrients are limiting. The form of the expression describing the rate of change of *X*_*i*_ *C* forces uptake of any non-limiting nutrients to be moderated:

2 $$ \frac{\mathrm{d}}{\mathrm{d} t}\;{X}_i C={\mu}_{\max }{X}_i{C}_{\mathrm{m}}\left[\left\{{X}_i C u>{ X C u}_{\min}\right\}{\theta}^{\beta}+\left\{{X}_i C u={ X C u}_{\min}\right\}\right]\cdot \frac{X_i}{X_i+{K u}_{x_i}}\cdot \frac{{\left(1-\frac{X_i C}{X_i{C}_{\mathrm{abs}}}\right)}^{Qh}}{{\left(1-\frac{X_i C}{X_i{C}_{\mathrm{abs}}}\right)}^{Qh}+{K}_{x_i}}-{ C u X}_i C $$

The first term on the right hand side (the nutrient uptake rate) contains logic expressions within the brackets that are governed by the outcome of Eq. (1). This expression tends to zero sigmoidally as *X*_*i*_ *C* approaches its absolute maximum value *X*_*i*_ *C*_abs_ at which point transport of the non-limiting nutrient ceases. The rate at which *X*_*i*_ *C* approaches *X*_*i*_ *C*_abs_ is regulated by the parameter *θ* ^*β*^. The explicit expressions for each stoichiometric variable modelled in this investigation are presented in Model_Info_equations in the Electronic Supplemental Material.

The carbon-specific growth rate *Cu* balances photosynthesis against losses through respiration. Total (depth integrated) photosynthetic activity, *PS*, in the water column is calculated by integrating the Smith equation (Smith 1936) over the system’s operational depth *τ* (assuming a homogeneous cell suspension) to obtain (Fasham et al. 2006):

3 $$ PS=\frac{Pqm}{k\tau}\left[ Ln\left(\frac{I_0\alpha ChlC}{Pqm}+\sqrt{1+{\left(\frac{I_0\alpha ChlC}{Pqm}\right)}^2}\right)-\mathrm{Ln}\left(\frac{I_0\alpha ChlC}{Pqm}{e}^{- k\tau}+\sqrt{1+{\left(\frac{I_0\alpha ChlC}{Pqm}{e}^{- k\tau}\right)}^2}\right)\right] $$

Here, *α* describes chlorophyll-specific photosynthetic efficiency at *I* = 0, *I*_0_ is the surface irradiance, *ChlC* is the mass ratio of chlorophyll to carbon, *Pqm* is the absolute maximum gross rate of photosynthesis (day^−1^; de facto the value of RuBisCO activity at the current nutrient status) and *k* is the attenuation factor of the culture and a function of *ChlC* and total algal C-biomass. Parameter *τ* is system depth. Variables are updated at each timestep to capture photoacclimation effects. The dynamics of photoacclimation (i.e. changes in the Chl/C quota with light and nutrient-status of the cells) are described so:

4 $$ \frac{\mathrm{d}\mathrm{ChlC}}{\mathrm{d} t}=\left\{\mathrm{ChlC}\le {\mathrm{ChlC}}_m\right\}\cdot {\mathrm{ChlC}}_m\cdot \mathrm{NPSCu}\cdot U m\cdot \left(1-\frac{PS}{Pqm}\right)\cdot \frac{\left(1-\frac{\mathrm{ChlC}}{{\mathrm{ChlC}}_m}\right)}{\left(1-\frac{\mathrm{ChlC}}{{\mathrm{ChlC}}_m}\right)+0.05}-\left\{\mathrm{ChlC}\ge 0.005\right\}\cdot \mathrm{ChlC}\cdot \left[ Cu+ Um\cdot \left(1- NCu\right)\right] $$

where

5 $$ Pqm=\left[ Um+ basres+{NC}_m\cdot Um\cdot \left( redco+1.5\right)\right]\cdot NPSCu $$

*NPSCu* is whichever is the lower of the N-, P- or (for diatom) Si-limited forms of Eq. 1; *basres* is basal respiration; *redco* is the cost for reducing nitrate to ammonium; 1.5 is the anabolic respiration cost in terms of gC per gN assimilated into biomass; *ChlC*_*m*_ is the maximum Chl/C (*ChlC*); *NCm* is the maximum cellular N/C; *Um* is the maximum growth rate (Flynn 2001).

Production of energy-rich C, considered as having potential as biofuel feedstocks, is related to the organism’s elemental C/N ratio and simulated using the method described by Flynn et al. (2012) through reference to the accumulation of excess-C that occurs during N-limited growth. This energy-rich C production provides an indicator of what returns are realistically possible, assuming that all such material is indeed suitable for conversion to biofuels. Taken together with the use of a maximum C/N value (minimum mass N/C = 0.05) that is at the extreme upper end of plausibility (Flynn 2008) to maximise the potential for accumulation of C-rich biofuels-feedstock metabolites, a high value for the slope of Chl-specific photosynthesis (*α* ^Chl^) to raise C-fixation, a low value of respiration to minimise production losses (see Flynn and Raven 2017), inevitably, the model outputs will represent overestimates in biofuels production. The stoichiometric values of the parameters used (see the ESM file Model_info_equations) are typical of experimentally derived values (e.g. Geider and LaRoche 2002). The sensitivity of the core models (and their prototypes) to the most critical parameters has been analysed previously (Flynn 2001, 2008).

### Simulation setup

Optimisations were performed using the proprietary evolutionary algorithms within Powersim Solver v.2 (Isdalstø, Norway) and simulations run within the Powersim Constructor v2.51 platform as we have used in our previous studies (Flynn et al. 2008; Flynn et al. 2012; Kenny and Flynn 2015, 2016). This optimisation software operates by searching through all pertinent input variable options to identify combinations of inputs that maximise production (of biomass or biofuels) set against certain criteria (such as minimising nutrient needs and space). From extensive prior experience (afore mentioned references), we know that biomass production is optimised through maintaining a nutrient-replete status, though for financial reasons minimising the concentration of residual (excess) nutrients in the culture. Conversely, optimising for biofuels production requires a level of nutrient stress to be developed in the cultures. Identifying the ideal conditions for optimised production in both scenarios is thus expedited by allowing the software to systematically adjust the input nutrient concentrations.

To take additional factors into account which had not been considered previously using a multi-nutrient mechanistic modelling approach (Kenny and Flynn 2015), additional operational scenarios were explored to those we have considered before. These compared continuous and discontinuous harvesting methods, with seasonal production optimised for each combination of latitude and depth (requiring different dilution rates over summer and winter). We assumed that for each production site, algal strains would be selected to grow optimally (realised growth rate close to *Um*) at the prevailing temperature regime; this could well involve the usage of different strains of microalgae during different seasons, especially for exploitation at higher latitudes. The parameter values explored are presented in Table 1. For presentation purposes, production was averaged over one calendar year to obtain a mean daily production rate.

**Table 1:** Model parameters varied for optimisation of areal productivity

Parameter	Description	Value range	Unit
max_depth	Depth	0.03–0.2	m
dil	Dilution rate	0.03–0.84	day^−1^
U_m_	Maximum growth rate	0.346–2.7	day^−1^
lat	Latitude	0–65	degrees
DINn_Conc	Nutrient-N concentration	6.17/12.35	gN m^−3^
DIP_Conc	Nutrient-P concentration	0.56/1.12	gP m^−3^
harvest_point	Harvest ratio day_*n*_/day_*n*+1_	1.03–1.99	Dimensionless

The full range of values explored in these simulations is given in the third column. Further explanation of the meaning and importance of these parameters is given in the Methods section (see also the ESM files Model_Info_schematic and Model_Info_equations, plus [30])

Maximum microalgal growth rate, *U*_*m*_, is the main driver of productivity (Flynn et al. 2012). The use of microalgal strains with a moderately high rate of *U*_*m*_ = 1.386 day^−1^ was investigated initially; this choice of *U*_*m*_ equates to a doubling of biomass over a 12:12 h light/dark cycle, giving growth rates attainable by the chlorophyte *Scenedesmus* (Lee 2001) and by diatoms (Lourenco et al. 2002). Additional simulations with varying *U*_*m*_ were also performed to better understand the broader potential for solar-powered microalgal biofuels production. The fastest microalgal growth rates measured experimentally typically fall in the region of 2.0 < *U*_*m*_ < 2.4 day^−1^ (Griffiths et al. 2011; Flynn and Raven 2017); as an extreme example, the marine diatom *Navicula acceptata* has been observed to undergo 3.8 doublings day^−1^ (Tadros and Johansen 1988) which gives a maximum growth rate *U*_*m*_ = 2.634 day^−1^. Accordingly, *U*_*m*_ was varied over a range of values signifying very low to exceptionally high growth (0.35 < U_*m*_ < 2.7 day^−1^); these are values that are consistent with cellular activities of RuBisCO (Flynn and Raven 2017). Production was optimised for the end product as either bulk biomass or as biofuels feedstocks using the evolutionary alogorithm in Powersim Solver v2.

The harvesting methods explored were continuous culture (akin to a chemostat), discontinuous culture with a prescribed daily harvesting/dilution frequency, and semi-automated harvesting triggered when growth plateaus (here set by parameter *harvest_point* as a minimal increase in biomass attained over 1 day, as may be achieved through monitoring culture absorbance). Discontinuous harvesting was configured to coincide with local dusk, to take advantage of a whole daylight’s worth of photosynthetic activity, before partial loss of algal-C through net cellular respiration during the dark phase (night). Harvesting other than using a continuous chemostat-like approach results in short periods of oscillation in nutrient status of the microalgae as the sudden input of significant volumes of fresh medium relieves nutrient stress. In such instances, the nutrient status of the microalgae typically re-entered quasi-steady state within the following light phase of growth. However, if the harvesting proportion was greater than ca. 50% on any one occasion, then (depending on the maximum growth rate and the time averaged dilution rate) re-establishment of quasi-steady state could take longer than one light–dark period.

For commercial production, optimising consumption of nutrients is important, both reflecting the cost of fertilisers (or of their recycling) and also to minimise the potential for eutrophication of local waterways in the event of spillage. Our previous studies have indicated that for maximum biomass productivity, N and P concentrations set to f/2 (Guillard and Ryther 1962) levels containing 12.35 mg N L^−1^ and 1.11 mg P L^−1^ (holding the N/P ratio constant throughout) work well in this regard (Kenny and Flynn 2015). While higher areal biomass yields (i.e. g biomass-C m^−2^) are inevitably obtained using higher nutrient concentrations, for commercial viability the areal rate of productivity (i.e. g biomass-C m^−2^ day^−1^) is the critically important factor. To ensure the required N-depletion develops to maximise biofuel production (g biofuel-C m^−2^ day^−1^), half-concentration nutrient levels (i.e. f/4) were found to be sufficient. For depths of 5 cm and less, f/2 levels proved more effective; the shallow depth permits higher nutrient levels to be used, giving higher biomass densities while still permitting N-source exhaustion and the consequential accumulation of surplus C for exploitation as biofuels. However, such conditions can only be achieved using specialised photobioreactors; open ponds require depths of ca. 20 cm to ensure adequate mixing (Tredici 2007; Ritchie and Larkum 2012).

Throughout, we assume that the culture system operates optimally from a logistic and engineering standpoint. Thus, we assume no mechanical breakdowns, all media transfers and harvesting attained extremely rapidly (modelled within a simulation timestep, of 12.5 min) and without wastage, no biomass loss through disease or cell disruption during culture, homogenous culture mixing with constant pH attained by CO_2_ input and matched O_2_ removal, no biofouling, etc. Although it is highly challenging to maintain such conditions in practice (and any deviations from the optimal state are, by definition, detrimental to production), considering such an idealised system allows us to bypass technological bottlenecks that may hamper the commercialisation of algae biofuel production and concentrate purely on physiological constraints. Our projections should thus be considered as representing the upper end of the productivity spectrum when operated over a whole year.

## Results

The model operates, as does biochemistry, with reference to carbon; results are thus given in terms of gC, not as dry weight. Transforms to dry weight units are considered in Discussion section.

**Fig. 1:**
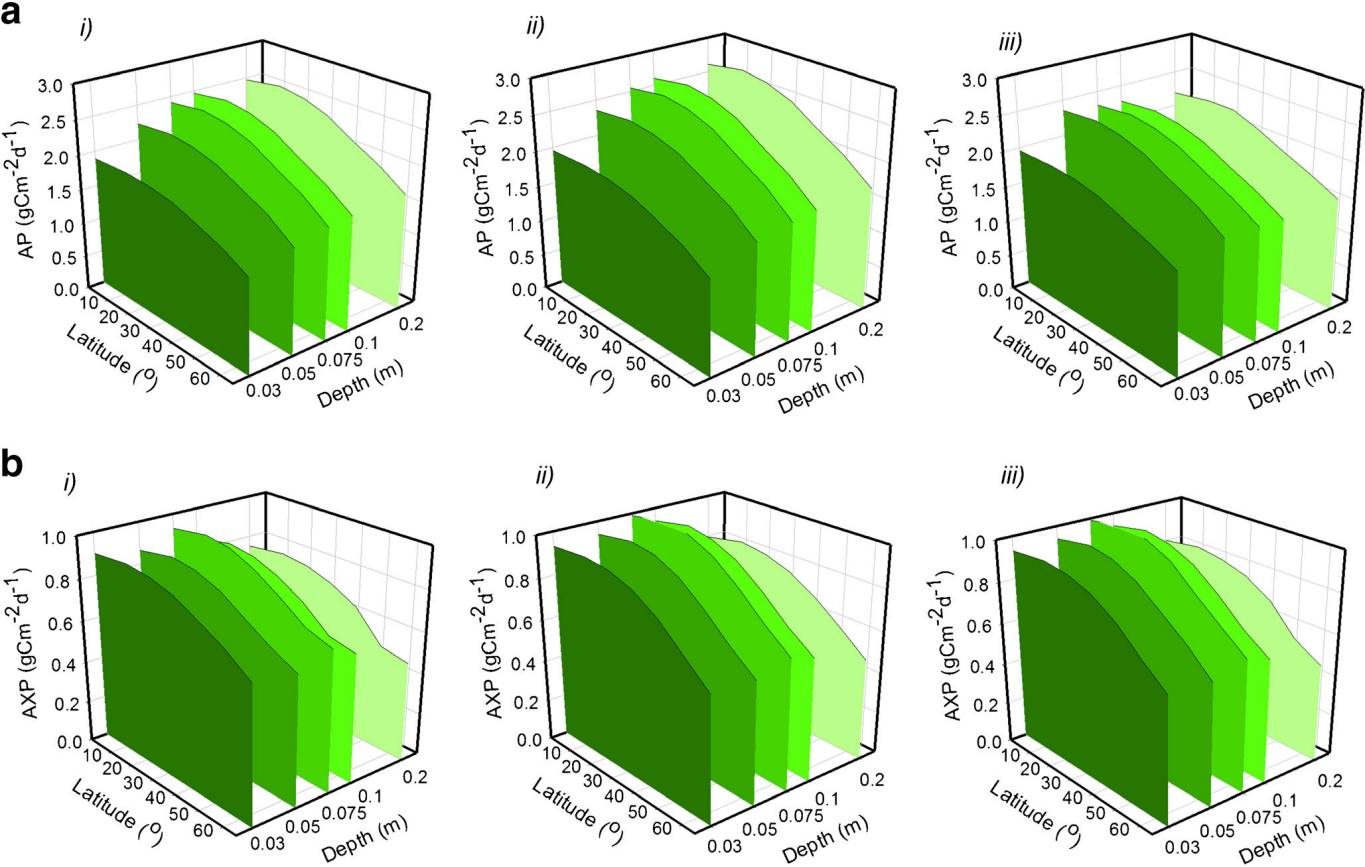
Biomass (**a**) and biofuel (**b**) production rates versus latitude and culture system depth achievable using microalgae with a maximum growth rate of 1.386 day^−1^. Three harvesting methods are compared; continuous culture (panels *i*), discontinuous culture with prescribed daily harvesting/dilution frequency (panels *ii*) and semi-automated harvesting triggered when growth plateaus (panels *iii*). Production of biomass (AP) and biofuel feedstocks (AXP) are averaged over one calendar year. The corresponding dilution rates, nutrient concentrations and volumetric productivities are presented in ESM Tables S1 to S4

**Fig. 2:**
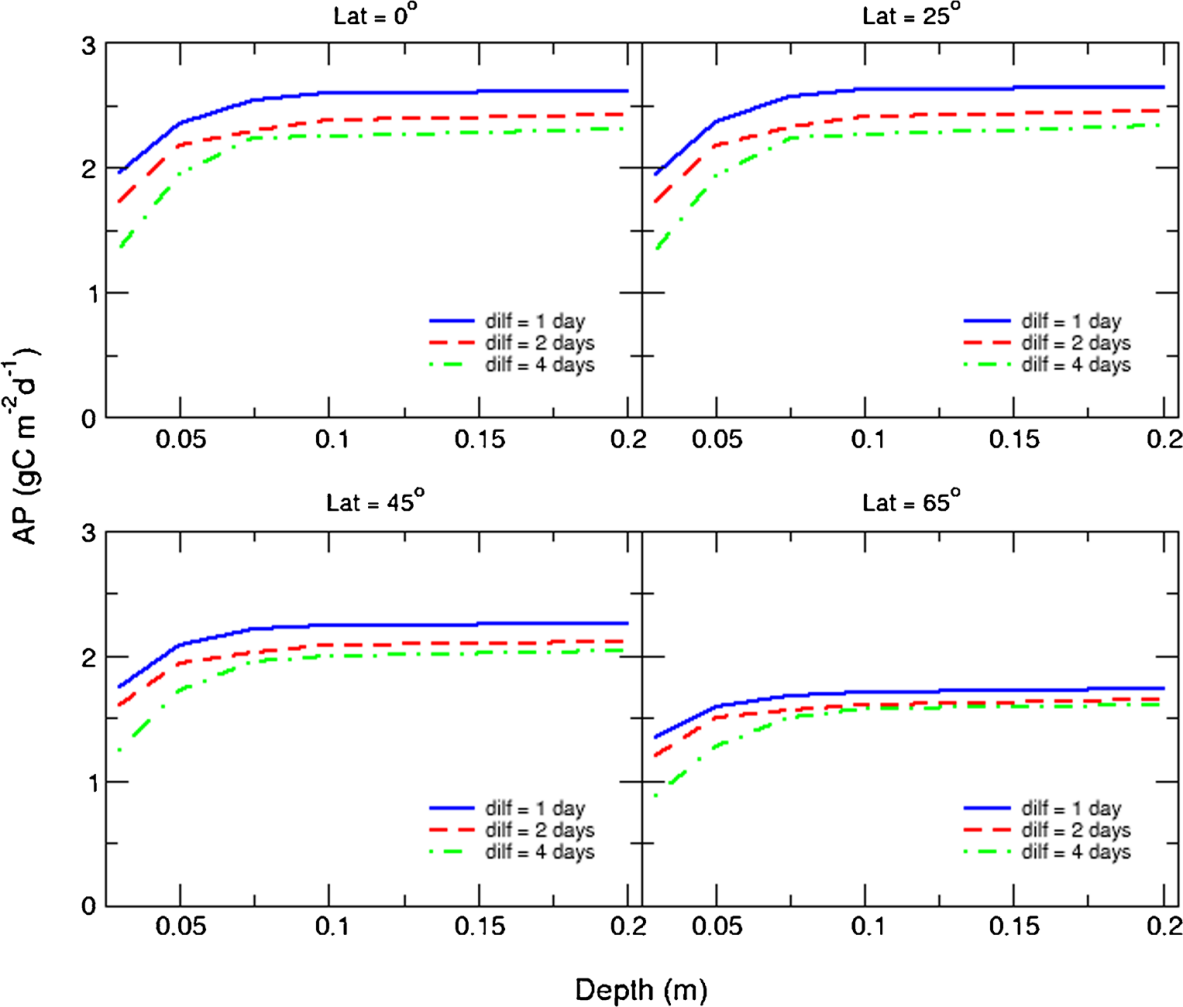
Optimised areal biomass production (AP) versus culture system depth for a sample of latitudes with prescribed harvest frequency set at *dilf* = 1, 2 and 4 days. Nutrient concentrations are at f/2 levels (see Methods section in the main text). The dilution rates required to achieve these production rates are outlined in Table S5. Production saturates at depths greater than 0.1 m due to self-shading effects in the culture system

**Fig. 3:**
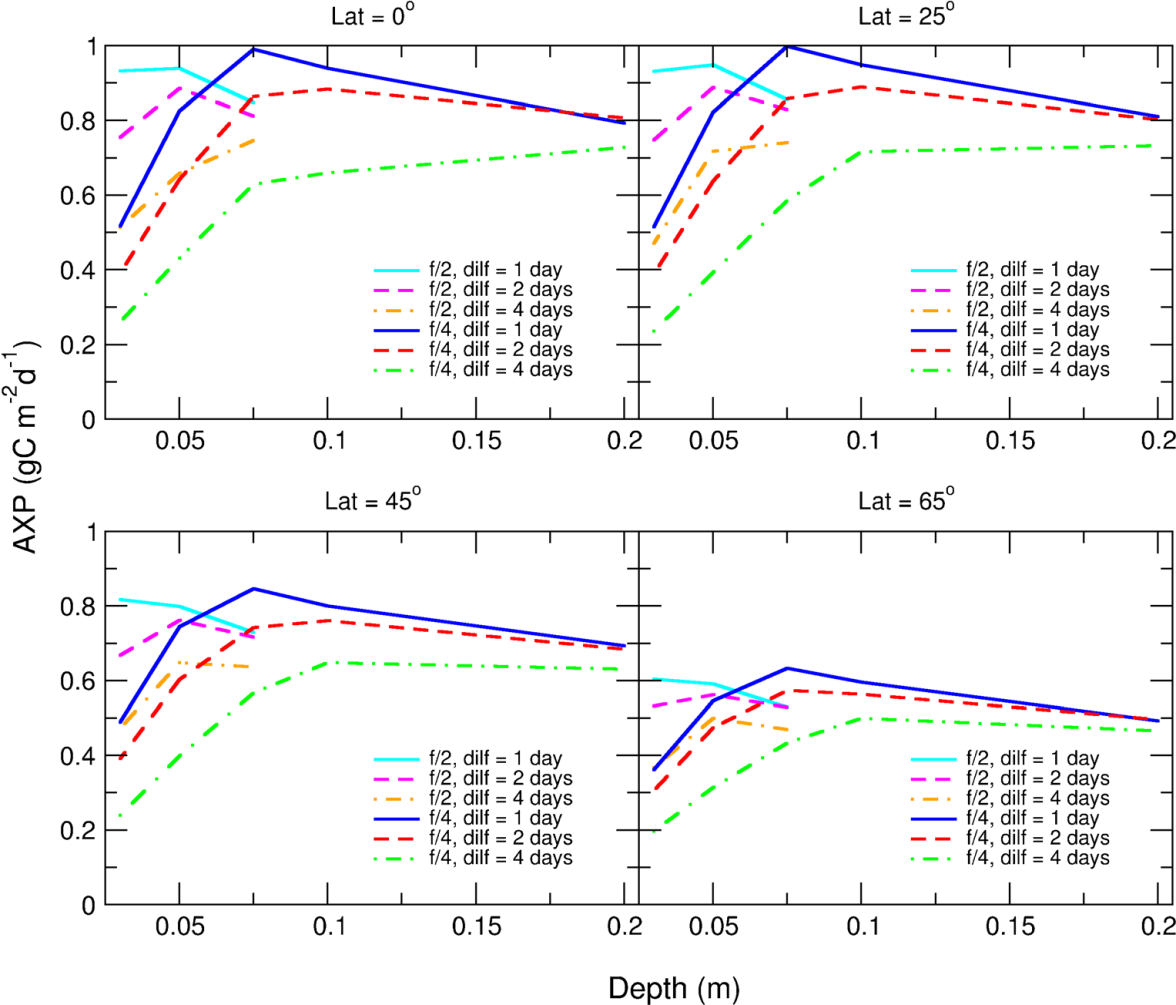
Optimised areal biofuel production (AXP) versus culture system depth for a sample of latitudes with prescribed harvest frequency set at *dilf* = 1, 2 and 4 days. Nutrient concentrations are at f/4 levels (see Methods section in the main text) for the majority of data points but also at f/2 levels at shallow optical depths. The dilution rates required to achieve these production rates are outlined in Table S5. Production saturates at depths greater than 0.1 m due to self-shading effects in the culture system

Figure 1 explores for microalgae of a typical microalgal maximum specific growth rate potential (*U*_*m*_ = 1.386 day^−1^) optimised areal production of biomass and biofuel feedstocks (identified hereafter as AP and AXP, respectively) for each combination of geographic location and bioreactor operational depth (i.e. pond depth, or ca. half tubular bioreactor diameter; Kenny and Flynn 2016). While these values varied little according to the choice of harvesting method (Fig. 1; see also Methods section), frequent discontinuous daily harvesting gave the best rates overall (Fig. 1, panels *ii*). Figures 2 and 3 illustrate how production declines with decreasing harvest frequency. AP saturated at bioreactor operational depths ≥10 cm, ranging as an annual average between 1.7 g biomass-C m^−2^ day^−1^ at high latitudes and 2.7 g biomass-C m^−2^ day^−1^ at tropical latitudes (see Fig. 1a). Optimal configurations for each harvesting mode, plus the resulting volumetric productions, are presented in Tables S1–S5 in the online ESM.

Optimising for biofuel feedstocks (Fig. 1b), peak AXP was achieved with growth in a 7.5 cm pond depth, ranging from 0.6 g biofuel-C m^−2^ day^−1^ at high latitudes to 1.0 g biofuel-C m^−2^ day^−1^ at tropical latitudes. However, in practice, ponds shallower than 15 cm are not viable due to the extreme difficulties in maintaining culture stability at such shallow depths (Tredici 2007; Ritchie and Larkum 2012). Assuming 20 cm to be the minimum practical operational depth, optimal AXP from a typical microalga growing in a solar-powered raceway at tropical latitudes becomes 0.83 g biofuel-C m^−2^ day^−1^ and, at higher latitudes, more typically 0.5 to 0.7 g biofuel-C m^−2^ day^−1^.

**Fig. 4:**
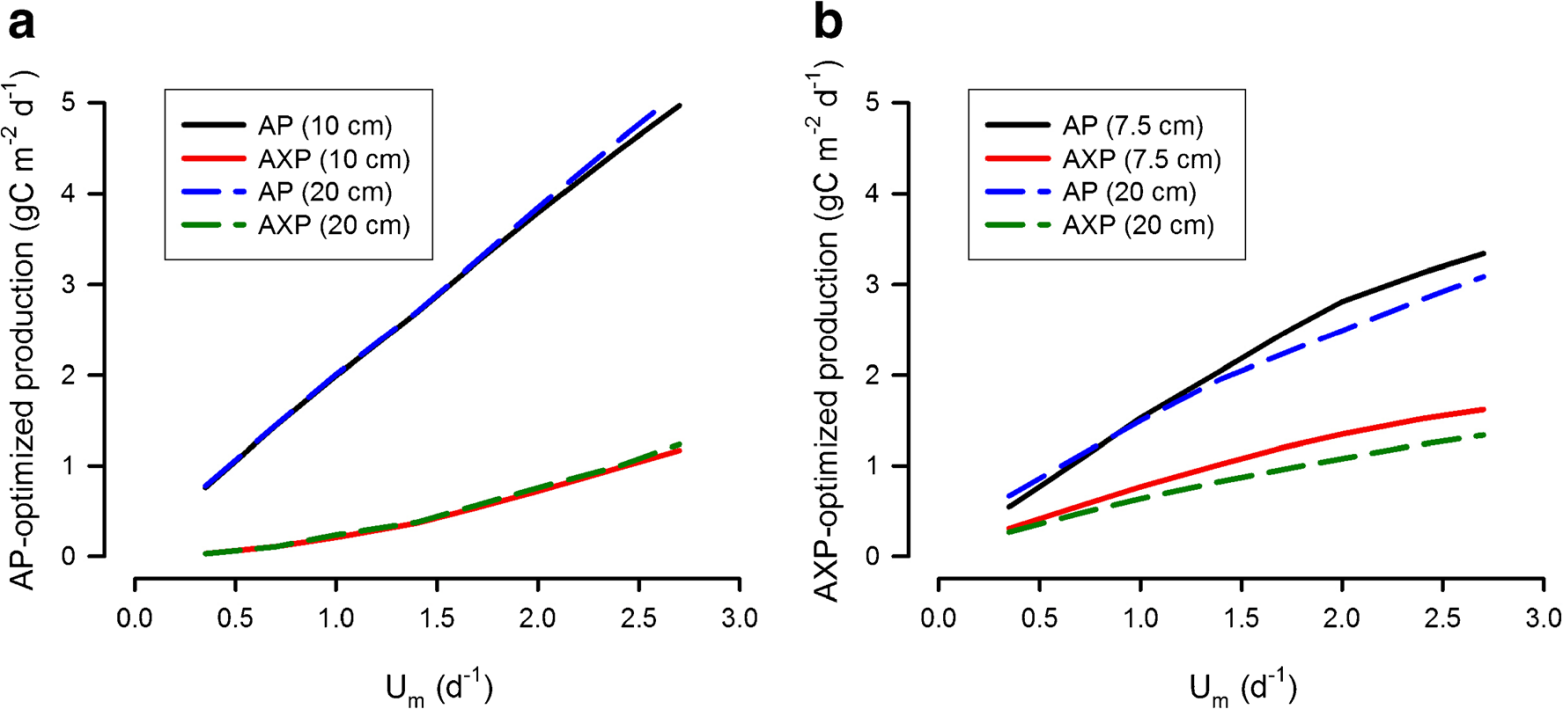
Comparison of optimised production for a range of maximum growth rates, *U*_*m*_. Areal production is optimised for biomass (AP; **a**) or biofuels (AXP; **b**). Culture system depths considered are those optimised for AP (10 cm) or for AXP (7.5 cm); also shown is production from systems operating within the minimum practical open pond depth of 20 cm

Optimal cultivation conditions were identified as occuring at 15° latitude, with discontinuous daily harvesting, using a 10 cm deep pond for AP and a 7.5 cm deep pond for AXP (noting that a 20 cm depth is required for both in commercial practice). Setting these conditions, the maximum specific growth rate, *U*_*m*_, was then varied to further explore production limits. Optimising for AP, biomass production increased linearly with *U*_*m*_ (Fig. 2a) and results using depths of 10 and 20 cm were virtually identical. AP for a microalgae strain with *U*_*m*_ = 2.7 day^−1^ (approaching four divisions a day, which is at the very upper extreme expected value (Flynn and Raven 2017]) averaged 5.1 g biomass-C m^−2^ day^−1^ over the year. For the most part, accumulation of energy-rich carbon was suppressed in these culture systems (because C-rich products are not synthesised during nutrient-replete high growth rates—Scott et al. 2010; Greenwell et al. 2010), but the proportion of biofuels-C to total C-biomass increased with increasing *U*_*m*_ (Fig. 4a); the ratio AXP/AP reached nearly 0.25 for *U*_*m*_ = 2.7 day^−1^.

Optimising for biofuels production (Fig. 4b, rather than biomass in Fig. 4a) with *U*_*m*_ = 2.7 day^−1^ gave an average AXP over the year of 1.6 g biofuel-C m^−2^ day^−1^ for a depth of 7.5 cm, falling to 1.3 g biofuel-C m^−2^ day^−1^ with the more realistic open pond depth of 20 cm (Fig. 4b). Assuming a diesel C-density of 720 gC L^−1^ (Miguel et al. 1998), these values equate respectively to 0.811 L and 0.66 L biodiesel m^−2^ year^−1^.

## Discussion

### Validity of the model

We start by placing the general performance of our simulation platform in the context of results claimed for real culture systems. Most production rates in the literature are given in terms of dry weight rather than gC; few researchers directly measure biomass-C. However, as the argument for algal biofuels is placed in the context of CO_2_ mitigation, quoting production in terms of C appears more appropriate. There is significant variation between conversion factors between C and dry weight. Transforms are in the range of 0.3–0.5 between cell C and dry weight (Heymans 2001; Geider and LaRoche 2002; Béchet et al. 2014), with the value expected to vary between species and also within species depending on the nutrient status. For a nutrient replete cell (low in carbohydrates and lipid), the ratio of C/dry weight will be lower. As maximum growth rates are attained under nutrient replete conditions, with protein-rich cells that are relatively poor in C, the lower transform value of 0.3 is thus the most appropriate when considering maximum biomass productivity.

The highest biomass production rates in the literature (e.g. Williams and Laurens 2010; Béchet et al. 2014) indicate values from growth in photobioreactors (PBRs) of as much as 30–40 g dw m^−2^ day^−1^, with extreme values of 60. However, production in the optically deeper ponds and raceways are much lower, at around 15 g dw m^−2^ day^−1^ (Williams and Laurens 2010). The maximum production rates projected by our simulations are 5.1 g biomass-C m^−2^ day^−1^. Applying the conversion factor of 0.3, we obtain a value from our simulations of pond or raceway style production of 17 g dw m^−2^ day^−1^. Although this value is fully in line with expectations, it is appropriate that we consider the significance of the disparity between our value and the highest values in the literature.

The vast bulk of literature values are from short-term culture runs, grown under summer conditions and/or in geographic locations of high irradiance; Quinn et al. (2012) present data for year round production showing the significant temporal variation expected at sites away from the equator over different months of the year. The highest productivities are also from cultures grown in PBRs. In contrast, our value represents a year-average, and obtained for optically deeper systems, in line with the need for truly massive cultures to be grown in open ponds or raceways. At worst, our annual productivity rates could be argued as being ca. 2–3-fold too low. Against that it is important to take into account the following:

1. We have assumed 100% reliability in engineering operations, including very rapid harvesting, completely effective nutrient cycling and continuous maintenance of system hygiene. This is a most improbable situation.
2. We assume no loss of production due to pests, disease or competitors (Borowitzka 2005; Flynn et al. 2017). Such an assumption, when applied to massive open pond systems extending over hundreds of hectares, appears unrealistic.
3. We assume no evolution of slower growth potential within the microalgal population. It is most likely that enforced growth at low rates in continuous-flow (chemostat-style) cultivation systems will result in the evolution of slower growing populations (Droop 1974), and the consequential loss of production as the maximum potential growth rate is a phenotypic characteristic that is a critical determinant of commercial productivity (Flynn et al. 2012).
4. We assume the maintenance of optimal temperature and pH, and thence of the availability of dissolved inorganic C. In reality, temperature will fluctuate over the seasons and over the day, while the continuous and adequate supply of CO_2_ is considered to represent a major restraint on location and operation of massive open systems (Williams and Laurens 2010).

Turning now to biofuels production, it is important to note that there is a significant difference in conditions required for effective biofuels production in comparison with biomass production (Kenny and Flynn 2015), and that areal production rates for biomass in systems optimised for biofuels production are far lower. This is as a consequence of effective production of high-C metabolites (carbohydrates and lipids) being promoted by growth into nutrient stress. There is thus an inverse relationship between growth rate and cellular lipid content (Shifrin and Chisholm 1981; Thompson et al. 1990), and between the cellular N/C and growth rate (Flynn 2008). Not only are biofuels-metabolites a fraction of total biomass-C, but the growth rate required for their optimised production is a fraction of the rate required to optimise for biomass production. To compound the challenges further, massive cultivation in raceways, that for practical reasons must be of ca. 20 cm depth (Tredici 2007; Ritchie and Larkum 2012), is ca. 2–3-fold lower than productivities possible in narrow bore (shallow light path) PBR systems.

In our model, we consider only the excess C deposition above the cellular C/N of N-replete cells; there are lipids in N-replete cells, associated with membranes (notably as unsaturated phospholipids), though these are less suited to conversion to biofuels (Greenwell et al. 2010). If one wished to include these fractions, then our predicted biodiesel production rates should be increased by ca. 20%. Set against this, we have assumed that 100% of the excess C is convertible with 100% efficiency to biodiesel, which is most unlikely. Importantly, our model also well replicates the extensive, and unique, data set of Quinn et al. (2012), with respect to areal production rates of biomass and biofuels-metabolites (Kenny and Flynn 2016).

Another test of our model’s performance is to consider the solar to chemical energy conversion efficiency. Assuming a maximum production rate using naturally occurring strains of microalgae of 8000 L ha^−1^ year^−1^ (achieved at tropical latitude 15°; Fig. 4b), and an energy density of each litre of biodiesel to be 35–40 MJ (L biodiesel)^−1^ (Williams and Laurens 2010), we compute a total areal energy production over the year of around 300 GJ ha^−1^ year^−1^, or about 1 Wm^−2^. The average sunlight power at this latitude (15°) over the period, as computed in our simulations and accounting for factors such as cloud cover and variations in solar elevation, is approximately 400 Wm^−2^. From the results of our analysis, the maximum solar to chemical energy conversion efficiency from algae is thus 0.25%, a value comparable with estimates of photosynthetic efficiency for lipid production of 0.31% (Ramachandra et al. 2013).

All things considered, our simulated biomass productivity rates appear to be representative of real potential rates in ponds or raceways, systems that represent the most plausible platform for the levels of production required to significantly mitigate against fossil fuel consumption. Our biofuels production rates also align with expectations and with expected solar conversion efficiencies.

### Determining the bounds of plausible long-term biofuels production

Assuming production could be continually maintained throughout the year (i.e. without failure of the culture system, and deploying algal strains that grew optimally throughout, with achieved specific growth rates close to their maximum), a biomass production rate of 2.7 g biomass-C m^−2^ day^−1^ (peak annually averaged AP in Fig. 1a) is attainable with organisms of a typical high growth rate (*U*_*m*_ = 1.386 day^−1^); this equates to just under 10 t biomass-C ha^−1^ year^−1^. This value obtained from our simulations falls in the mid to upper range of rates achieved in real pond systems (Jimanez et al. 2003; de Schamphelaire and Verstraete 2009; Crowe et al. 2012), assuming a C/dry weight ratio between 0.3 and 0.5 (Heymans 2001; Geider and LaRoche 2002), and is also consistent with outputs from other calculations (Ritchie and Larkum 2012) (see also Introduction section). Maximum production is achieved at around latitude 15°, reflecting a balance between light levels during the day, cloud cover and set against duration of the daylight hours. In darkness, respiration consumes a proportion of the newly fixed C, while the resource expensive CO_2_-fixing enzyme RuBisCO (and all other components of the photosynthetic apparatus) is essentially dormant. In consequence, productivity levels at medum-to-high latitudes during summer may be quite respectable (Kenny and Flynn 2015), though annual productivities are low, suppressed by long winter nights. It is unlikely that truly massive culture systems at high latitudes could lay unused over winter without incurring significant re-start and close-down costs to enable summer-only operations.

Assuming *U*_*m*_ at 2.7 day^−1^ (Fig. 4a), a value at the extreme end of growth rates that are plausible in long-term growth systems based on RuBisCO activity (Flynn and Raven 2017), raises areal biomass production to 5.1 g biomass-C m^−2^ day^−1^. This value equates to 50–60 t dw ha^−1^ year^−1^ using the C/dw assumption above, a value that is similar to the higher reported algal biomass production rates (Moheimani and Borowitzka 2006; tabulated in supplementary information within Béchet et al. 2014). While there are higher values (Béchet et al. 2014), these appear as exceptions and there is insufficient corroboration that such rates could be achieved or maintained in the open ponds operated for mass biofuels (rather than biomass) production operated over a year-long cycle. A rate of production as high as 9 g lipid m^−2^ day^−1^ can be calculated from the work of Rodolfi et al. (2009). These authors used a mid-summer production run with a tubular bioreactor. They also used a two-phase process in which a nutrient-replete biomass was then subjected to a nutrient deplete state. Such a two-phase approach at least doubles the space required, so the areal productivity in practice is halved. Our model projects production rates approaching 4 g biofuels-C m^−2^ day^−1^ operating only during a long-day length summer light scenario and with a small-bore bioreactor (see Flynn et al. 2012, noting projections in that work are for a 12 h:12 h light/dark cycle). Extrapolating short-duration, sub-pilot-level studies conducted in PBRs, such as that by Rodolfi et al. (2009), to the potential for massive industrial scale production over a whole year in open ponds is problematic in the extreme.

Our simulated optimised AXP peaks at 1.0 g biofuel-C m^−2^ day^−1^ (Fig. 1b) when using a moderately fast growing microalgal strain (*U*_*m*_ = 1.386 day^−1^). Assuming a carbon density of 720 gC L^−1^ (typical of diesel fuels; Miguel et al. 1998), this would equate to an annual areal biofuel production rate of, at best, 5200 L ha^−1^ year^−1^. However, operating with a more realistic minimum viable pond depth of 20 cm (cf. Tredici 2007; Ritchie and Larkum 2012), peak productivity falls to 0.83 g biofuel-C m^−2^ day^−1^ (see Table 2), equating to 4200 L ha^−1^ year^−1^ (and, more typically, 2500 to 3500 L ha^−1^ year^−1^ at higher latitudes). Repeating this analysis assuming a potential microalgal autotrophic growth rate at the upper extreme of plausibility (*U*_*m*_ = 2.7 day^−1^; Flynn and Raven 2017), using a 7.5 cm reactor depth (Fig. 4b), gives a peak of 1.6 g biofuel-C m^−2^ day^−1^. This places an absolute limit on long-term solar-powered biodiesel production using a natural microalgal strain at approximately 8000 L ha^−1^ year^−1^. Growth using such a shallow depth requires use of specialised photobioreactors which would be inpracticable for a truly massive culture system; assuming 20-cm-deep industrial-scale raceways lowers peak productivity to 1.3 g biofuel-C m^−2^ day^−1^, or approximately 6500 L biodiesel ha^−1^ year^−1^. There are, however, allied commercial constraints associated with maintaining such a production rate that must also be considered.

**Table 2:** Nutrient consumption for biomass and biofuel production at various latitudes

Biomass production					
Depth 10 cm					
Lat	dil_w (day^−1^)	dil_s (day^−1^)	AP (gC m^−2^ day^−1^)	N use (gN kgC^−1^)	P use (gP kgC^−1^)
0	0.39	0.39	2.60	185	17
15	0.37	0.41	2.69	179	16
25	0.35	0.42	2.63	180	16
35	0.32	0.43	2.49	186	17
45	0.28	0.43	2.25	195	18
55	0.21	0.44	2.02	198	18
65	0.12	0.45	1.71	206	19
Depth 20 cm					
Lat	dil_w (day^−1^)	dil_s (day^−1^)	AP (gC m^−2^ day^−1^)	N use (gN kgC^−1^)	P use (gP kgC^−1^)
0	0.39	0.39	2.61	368	33
15	0.37	0.41	2.70	356	32
25	0.35	0.42	2.64	359	33
35	0.32	0.43	2.50	370	33
45	0.28	0.43	2.25	389	35
55	0.21	0.44	2.03	395	36
65	0.08	0.45	1.73	377	34
Biofuel production					
Depth 7.5 cm					
Lat	dil_w (day^−1^)	dil_s (day^−1^)	AXP (gC m^−2^ day^−1^)	N use (gN L^−1^)	P use (gP L^−1^)
0	0.29	0.28	0.99	96	9
15	0.28	0.31	1.02	96	9
25	0.27	0.31	1.00	97	9
35	0.24	0.32	0.94	99	9
45	0.2	0.31	0.85	100	9
55	0.15	0.31	0.73	105	9
65	0.1	0.32	0.63	110	10
Depth 20 cm					
Lat	dil_w (day^−1^)	dil_s (day^−1^)	AXP (gC m^−2^ day^−1^)	N use (gN L^−1^)	P use (gP L^−1^)
0	0.12	0.13	0.79	140	13
15	0.13	0.14	0.83	144	13
25	0.12	0.14	0.81	142	13
35	0.11	0.14	0.77	144	13
45	0.09	0.14	0.69	148	13
55	0.05	0.14	0.60	141	13
65	0.03	0.13	0.49	145	13

Nitrogen and phosphorous use are calculated for each latitude from the winter and summer dilution rates (dil_w, dil_s) using nutrient levels described in Methods section. Depths correspond to the optimal depths for enhanced production (areal biomass production, AP at 10 cm; areal biofuels production, AXP, at 7.5 cm) and also the minimum practical depth for large open ponds (20 cm). Nutrient use is quoted per kilogramme of biomass (AP) or per litre of biodiesel (AXP) produced

### Revisiting commercial viability

In the Introduction section, we noted that published life cycle analyses (LCAs) exploring the commercial viability of microalgal biofuels considered a range of productivity scenarios, with only the highest values (ca. 30 g biofuel-C m^−2^ day^−1^) considered as leading to a positive outcome. We now use our results to re-appraise the LCAs conducted by Sun et al. (2011) and Davis et al. (2011). Our simulations show that the scope for autotrophic microalgal production cannot be increased beyond even the low/baseline case scenarios used in these LCAs. Even extrapolating to year-round pond production from claims made for short-term tubular bioreactor, for a rate of ca. 9 g biofuels-C m^−2^ day^−1^ (Rodolfi et al. 2009), gives scant cause for optimism as this value is still at the bottom end of the production range considered by Sun et al. (2011), and shown by them as being inadequate on commercial grounds. And all the time it is necessary to recall that significant extra space is occupied by the vital ancillary operations associated with the massive preparation of fresh media, harvesting, and recovery of nutrients and water in culture systems that must occupy many hundreds of hectares.

Venturing to the extreme of maximum claimed biomass productivities (ca. 60 g dw m^−2^ day^−1^) with maximum lipid content (60%) (see Williams and Laurens 2010), a situation that is physiologically implausible given the inverse relationship between growth rate and lipid content, and the highest conversion of C to dry weight (0.5), we achieve a value for biofuels productivity of (60 × 0.5 × 0.6) 18 g biofuel-C m^−2^ day^−1^. Even this value is far below the commercial viability threshold of 30 g biofuel-C m^−2^ day^−1^.

An alternative to growing microalgae solely for biofuels is to operate an integrated biorefinery operation, in which biofuels represent but one of many potential products (Greenwell et al. 2010; Wijffels and Barbosa 2010). From a commercial perspective, it is then necessary to consider the trade-off in the value of biomass versus that for biofuels. This is complicated by the fact that the areal production of biofuels does not increase in simple proportion to areal production of biomass (AXP vs. AP; Fig. 4). If biofuels optimisation takes priority over that for biomass production, then our simulations suggest that, as *U*_*m*_ increases, more and more biomass production potential must be sacrificed to eke out diminishing gains in biofuel productivity (compare AP and AXP in Fig. 4a against those in Fig. 4b). For example, when optimising for AXP versus AP at a growth rate of *U*_*m*_ = 2.7 day^−1^, our simulations suggest that 40% of (potentially very high-value) biomass production capacity would have to be forfeited for a <10% gain in low-value biofuels production (Fig. 4a, b).

However, an inability to achieve simple commercial viability in itself does not itself mean that exploiting microalgae for biofuels will not become energetically or sustainably attractive; we now turn to these aspects.

### Energetics, sustainability and logistical constraints

It is possible to assess potential energy returns by reconsidering published results from existing LCAs in consequence of the results from our simulations. The biomass productivity rates predicted by our simulations (Fig. 4) fall within the range of the ‘low-production’ scenario considered by Sills et al. (2013); such values permit only negative net returns on energy supplied for harvesting and extraction, even under the most optimistic limits of their sensitivity analysis. Truly massive cultivation of microalgae, over areas of many hundreds of hectares, cannot be conducted in high-performance, low-depth, high-cost, photobioreactors built using glass or plastic tubing. Massive cultivation requires cheap open pond (raceway) systems. Within any culture system, an important commercial consideration in optimising microalgal production is the need to balance areal and volumetric production (i.e. balancing land costs with de-watering costs) while minimising resource consumption. While high biomass ponds provide for lower dewatering costs per mass of algae, to obtain a good production rate of biofuels feedstocks an optically thinner suspension is required (as N and not light must limit growth, so leading to accumulation of C-rich metabolites). This is particularly problematic for commercial biofuels production, where high-cost nutrients (mainly inorganic N and P) must be recycled, thus placing an additional demand on space and energy.

To better understand these issues, we analysed nutrient use for optimised AP and AXP within a system operated in discontinuous (daily harvest) mode (see Table 2). The most conservative use of resources for optimised AP averaged 190 gN (kgC-biomass)^−1^ and 17 gP (kgC-biomass)^−1^. For optimised AXP (Table 2), N and P usage averaged 100 gN (L biodiesel)^−1^ and 9 gP (L biodiesel)^−1^; however, using the more realistic 20-cm-deep raceways, inputs of 144 gN (L biodiesel)^−1^ and 13 gP (L biodiesel)^−1^ are required. These consumption figures are of the same order as those estimated using (fixed) stoichiometric arguments for biomass production (Wijffels and Barbosa 2010; Pate et al. 2011; Yang et al. 2011b, b), though our values for nutrient usage are specifically generated for biofuel production obtained using an acclimative, variable stoichiometric model.

To place these nutrient demands into context, we consider as an example the specific challenge set by the European Commission, through its Renewable Energy Directive, to supply 10% of transport energy in member states by renewable sources by 2020 while at the same time remaining environmentally sustainable and economically viable (Soundararajan and Thomson 2013). Taking growing conditions typical of Europe (i.e. around latitude 45°), our nutrient demand calculations (see Table 2) show that a biodiesel production of 3500 L ha^−1^ year^−1^ in 20-cm-deep raceways demands 148 gN (L biodiesel)^−1^ and 13 gP (L biodiesel)^−1^. For algal biofuels to replace 10% of the 350 billion litres of transport fuel consumed in the EU each year (European Commission 2014) would thus require growth ponds totalling an area of ca. 10 million ha; this is approximately equivalent to 6% of Europe’s agricultural hectarage (Langeveld et al. 2014) and is 20 times larger than the total area of uncontaminated brownfield land available for redevelopment across Europe (Maliene et al. 2012). Additionally, such production would consume, and hence require recycling of, 5 million tonnes of N and 455,000 t of P annually. Put in perspective, the latter equates to nearly half of the P (in the form of P_2_O_5_) used to fertilise Europe’s terrestrial crops each year (Tóth et al. 2014).

Taken all together, merging our results into previous studies of commercial viability and energetic sustainability leads us to cast significant doubt over a plausible role of microalgae for biofuels production to replace fossil fuels. Although some reports indicate potential for very high biomass production rates (tabulated in supplementary information within Béchet et al. 2014), it is important to appreciate that such biomass productivities cannot be simply extrapolated to biofuels productivity because of the aforementioned physiological constraints. Production rates also need to be averaged over the year; while results from ponds run at higher latitude for ca. 3 months in summer may be suggestive of higher production potential, they would not do so over the whole year taking into account lower winter irradiance levels. This challenge is amply illustrated in the data of Quinn et al. (2011), a data set to which our model fits (Kenny and Flynn 2016). Yearly energy-rich carbon production rates one order of magnitude greater than our predictions would be required to make algae-derived biofuels competitive. One route forwards is to consider some form of genetic or allied biological modification to enhance production.

### Countering physiological constraints on autotrophic microalgal biofuels production

If algal biofuels are to become viable then, first and foremost, the growth rates of microalgae need to be raised very significantly above those we currently observe as plausible for prolonged cultivation (Flynn and Raven 2017). The challenge thus appears undeniably a biological one, rather than one of engineering systems for enhancing cultivation and harvesting.

Enhancing autotrophic microalgal growth requires emphasis to be placed on minimising self-shading caused by photoacclimation and increasing the net activity of RuBisCO so that maximum rates of photosynthesis are increased. Minimising self-shading has been the target of genetic modification approaches for some time (Beckmann et al. 2009; Melis 2009), but the change cannot be stable as any microalgal cells that (through natural variability) express enhanced photoacclimation will outcompete such engineered strains for photons. Increasing the net activity of RuBisCO through manipulations has been the subject of considerable effort for crop plants (Carmo-Silva et al. 2015) in which the structural complexities of these higher plants, the low concentration of atmospheric CO_2_ and the plant’s need for water, plus RuBisCO operational integrity when subjected to significant and rapid changes in temperature, have all been identified as important factors. In basic terms, for microalgal cellular RuBisCO activity to be increased requires raising the specific catalytic activity (*K*_cat_) and/or that the percentage of cellular protein as RuBisCO is increased (Flynn and Raven 2017). The latter conflicts with the need to minimise cellular N (and thence N/C) content for high biofuels, while doubts have been raised over there being any adaptive or energetic advantage in maintaining a higher value of *K*_cat_ (Tcherkez et al. 2006); in vivo expression of that enhanced *K*_cat_ in high density culture is also doubtful (Flynn and Raven 2017).

Interestingly, the review of Carmo-Silva et al. (2015) does not say what level of enhanced net C-fixation may be possible for crop plants through manipulations of RuBisCO. Given that in the absence of light limitation, this enzyme constrains growth through C-fixation we can, however, explore just how high activities would need to be raised to achieve the required level of microalgal production for biofuel viability (assuming all else remains equal). Our previous analysis of the optimum configuration of a ‘GM-biofuels-microalgae’ (Flynn et al. 2012) coupled with the operational analysis presented here indicates that an increased rate of cellular growth by ca. 5-fold would be required to at least approach viability with respect to areal productivity. From the analysis of Flynn and Raven (2017), such an increase in microalgal growth rate appears implausible without a de facto artificial replacement for RuBisCO, which is the single most important and abundant enzyme on Earth.

### Outlook

The overarching conclusion to draw from merging the results from our simulations with previously published commercial and energetic-facing LCAs is that solar-powered cultivation of natural algae strains exclusively for biofuels at the levels required to make a significant impact on fossil fuel usage appears to be neither commercially viable nor to provide positive energy returns. Even set within a biorefinery concept, with optimisation for biomass growth using a fast-growing strain (Fig. 4a) and with energy-rich components produced sub-optimally, the prospects for production of significant volumes of biofuel at a positive commercial value appear slight. In the long term, fast growing traits (required to maximise potential biofuels production) are likely to be selected against under the enforced slow microalgal growth (Flynn 2009; Schaum and Collins 2014) required to maximise biofuels production in N-limited systems. Thus, commercially at least, it is reasonable to discount the very highest biofuel production rates claimed altogether, even though they might be achievable in practice over the short term under highly tuned conditions.

The above mentioned are not technological barriers in engineering; they are biological constrains and are set mainly by two fundamental characteristics of algal physiology (namely photoacclimation and RuBisCO activity). Very significant enhancements to microalgal productivity (i.e. approaching 5-fold) through GM techniques are needed to overcome these constraints. However, from the forgoing, even this enhancement does not guarantee commercial or sustainable success, and that is before considering the potential environmental risks posed by the inevitable escape to the wild of such organisms grown in vast open ponds (Flynn et al. 2012). Pragmatic considerations surrounding the sourcing of CO_2_, N and P fertilisers, and the need for a rapid and 100% recirculation of those fertilisers adds an additional twist to the logistic challenge.

If operating systems using a GM microalgae (assuming that would be accepted within massive open pond systems), such that the hectarage required in the above mentioned example of European biofuels production was decreased by ca. 5-fold (to 2 million ha), the flux of N and P fertiliser around the system would remain at many hundreds of thousands of tonnes annually. Even deploying some form of coupled photovoltaic solar farm with LED-lit PBR installations, allowing continuous irradiance and a relatively bio-secure platform for GM microalgae, would require that fertiliser flow. And then there is the irony that to achieve this productivity the input of a commensurate quantity of CO_2_ to balance removal during microalgal C-fixation would still be required, CO_2_ which is most readily sourced from heavy industry (such as steel manufacture or fossil-fuel burning power stations). As Williams and Laurens (2010) allude, this demand for CO_2_ alone places a very significant, and potentially critical, burden upon the viability of the whole microalgal biofuels agenda.

## Electronic supplementary material

ESM 1 (DOC 300 kb)

ESM 2 (XLS 48 kb)

ESM 3 (XLS 48 kb)

ESM 4 (XLS 44 kb)
