# Supplementary material for: Physiology limits commercially viable photoautotrophic production of microalgal biofuels
Source: J Appl Phycol. 2017 Jul 13;29(6):2713–27. doi: 10.1007/s10811-017-1214-3 (PMC5705747; doi:10.1007/s10811-017-1214-3)
Supplement: Supplementary file 1 — (DOC 300 kb) [file 10811_2017_1214_MOESM1_ESM.doc]

# **Electronic Supporting Material**

**Physiology limits commercially viable photoautotrophic production of microalgal biofuels**

Philip Kenny & Kevin J Flynn

***Table S1.*** *Continuous dilutions (D) at various culture system depths for optimised biomass and biofuel production illustrated in figure 1, panels i. Areal and volumetric biomass production (AP, VP) have full units of g biomass-C m^-2^ d^-1^. Areal and volumetric biofuels production (AXP, VXP) have full units of g biofuels-C m^-2^ d^-1^.*

|  |  | Biomass | | | |  | Biofuel | | | |
| --- | --- | --- | --- | --- | --- | --- | --- | --- | --- | --- |
| Latitude  (°) | Depth  (m) | D (d^-1^)  (Winter) | D (d^-1^)  (Summer) | AP  (gCm^-2^d^-1^) | VP  (gCm^-3^d^-1^) |  | D (d^-1^)  (Winter) | D (d^-1^)  (Summer) | AXP  (gCm^-2^d^-1^) | VXP  (gCm^-3^d^-1^) |
| 0 | 0.03 | 0.37 | 0.37 | 1.96 | 65.2 |  | 0.32 | 0.31 | 0.93 | 31.1 |
|  | 0.05 | 0.39 | 0.39 | 2.36 | 47.1 |  | 0.24 | 0.23 | 0.94 | 18.8 |
|  | 0.075 | 0.39 | 0.39 | 2.55 | 34.0 |  | 0.29 | 0.28 | 0.99 | 13.2 |
|  | 0.1 | 0.39 | 0.39 | 2.60 | 26.0 |  | 0.23 | 0.23 | 0.94 | 9.4 |
|  | 0.2 | 0.39 | 0.39 | 2.61 | 13.1 |  | 0.12 | 0.13 | 0.79 | 4.0 |
| 15 | 0.03 | 0.35 | 0.38 | 1.98 | 65.9 |  | 0.3 | 0.32 | 0.95 | 31.5 |
|  | 0.05 | 0.37 | 0.41 | 2.42 | 48.3 |  | 0.22 | 0.24 | 0.97 | 19.3 |
|  | 0.075 | 0.37 | 0.41 | 2.63 | 35.0 |  | 0.28 | 0.31 | 1.02 | 13.6 |
|  | 0.1 | 0.37 | 0.41 | 2.69 | 26.9 |  | 0.23 | 0.25 | 0.97 | 9.7 |
|  | 0.2 | 0.37 | 0.41 | 2.70 | 13.5 |  | 0.13 | 0.14 | 0.83 | 4.1 |
| 25 | 0.03 | 0.34 | 0.39 | 1.95 | 65.0 |  | 0.29 | 0.34 | 0.93 | 31.1 |
|  | 0.05 | 0.35 | 0.42 | 2.37 | 47.5 |  | 0.22 | 0.26 | 0.95 | 19.0 |
|  | 0.075 | 0.35 | 0.42 | 2.57 | 34.3 |  | 0.27 | 0.31 | 1.00 | 13.3 |
|  | 0.1 | 0.35 | 0.42 | 2.63 | 26.3 |  | 0.22 | 0.25 | 0.95 | 9.5 |
|  | 0.2 | 0.35 | 0.42 | 2.64 | 13.2 |  | 0.12 | 0.14 | 0.81 | 4.1 |
| 35 | 0.03 | 0.32 | 0.4 | 1.88 | 62.6 |  | 0.27 | 0.34 | 0.89 | 29.8 |
|  | 0.05 | 0.33 | 0.42 | 2.27 | 45.4 |  | 0.2 | 0.26 | 0.89 | 17.9 |
|  | 0.075 | 0.32 | 0.43 | 2.44 | 32.6 |  | 0.24 | 0.32 | 0.94 | 12.6 |
|  | 0.1 | 0.32 | 0.43 | 2.49 | 24.9 |  | 0.19 | 0.26 | 0.89 | 8.9 |
|  | 0.2 | 0.32 | 0.43 | 2.50 | 12.5 |  | 0.11 | 0.14 | 0.77 | 3.8 |
| 45 | 0.03 | 0.27 | 0.4 | 1.74 | 58.0 |  | 0.23 | 0.34 | 0.82 | 27.3 |
|  | 0.05 | 0.28 | 0.43 | 2.07 | 41.5 |  | 0.16 | 0.25 | 0.80 | 16.0 |
|  | 0.075 | 0.28 | 0.43 | 2.21 | 29.4 |  | 0.2 | 0.31 | 0.85 | 11.3 |
|  | 0.1 | 0.28 | 0.43 | 2.25 | 22.5 |  | 0.16 | 0.26 | 0.80 | 8.0 |
|  | 0.2 | 0.28 | 0.43 | 2.25 | 11.3 |  | 0.09 | 0.14 | 0.69 | 3.5 |
| 55 | 0.03 | 0.24 | 0.41 | 1.58 | 52.7 |  | 0.18 | 0.35 | 0.71 | 23.7 |
|  | 0.05 | 0.21 | 0.44 | 1.88 | 37.5 |  | 0.12 | 0.24 | 0.69 | 13.8 |
|  | 0.075 | 0.21 | 0.44 | 1.98 | 26.5 |  | 0.15 | 0.31 | 0.73 | 9.8 |
|  | 0.1 | 0.21 | 0.44 | 2.02 | 20.2 |  | 0.12 | 0.25 | 0.69 | 6.9 |
|  | 0.2 | 0.21 | 0.44 | 2.03 | 10.1 |  | 0.05 | 0.14 | 0.60 | 3.0 |
| 65 | 0.03 | 0.14 | 0.44 | 1.34 | 44.7 |  | 0.12 | 0.34 | 0.60 | 20.1 |
|  | 0.05 | 0.12 | 0.45 | 1.58 | 31.7 |  | 0.08 | 0.23 | 0.59 | 11.8 |
|  | 0.075 | 0.12 | 0.45 | 1.67 | 22.3 |  | 0.1 | 0.32 | 0.63 | 8.5 |
|  | 0.1 | 0.12 | 0.45 | 1.71 | 17.1 |  | 0.08 | 0.21 | 0.60 | 6.0 |
|  | 0.2 | 0.08 | 0.45 | 1.73 | 8.7 |  | 0.03 | 0.13 | 0.49 | 2.5 |

***Table S2.*** *Discontinuous dilutions (D) with prescribed daily harvest at various culture system depths for optimised biomass and biofuel production illustrated in figure 1, panels ii. Areal and volumetric biomass production (AP, VP) have full units of g biomass-C m^-2^ d^-1^. Areal and volumetric biofuels production (AXP, VXP) have full units of g biofuels-C m^-2^ d^-1^.*

| Latitude  (°) | Depth  (m) | D (d^-1^)  (Winter) | harvest_point  (Winter) | D (d^-1^)  (Summer) | harvest_point  (Summer) | AP  (gCm^-2^d^-1^) | VP  (gCm^-3^d^-1^) |
| --- | --- | --- | --- | --- | --- | --- | --- |
| 0 | 0.03 | 0.37 | 1.77 | 0.37 | 1.8 | 1.96 | 65.2 |
|  | 0.05 | 0.39 | 1.68 | 0.39 | 1.71 | 2.36 | 47.1 |
|  | 0.075 | 0.58 | 1.65 | 0.58 | 1.65 | 2.30 | 30.7 |
|  | 0.1 | 0.66 | 1.61 | 0.66 | 1.62 | 2.28 | 22.8 |
|  | 0.2 | 0.78 | 1.57 | 0.77 | 1.48 | 2.19 | 11.0 |
| 15 | 0.03 | 0.37 | 1.65 | 0.39 | 1.73 | 1.97 | 65.7 |
|  | 0.05 | 0.37 | 1.72 | 0.41 | 1.78 | 2.41 | 48.2 |
|  | 0.075 | 0.55 | 1.6 | 0.32 | 1.47 | 2.37 | 31.5 |
|  | 0.1 | 0.65 | 1.68 | 0.67 | 1.5 | 2.33 | 23.3 |
|  | 0.2 | 0.81 | 1.55 | 0.81 | 1.55 | 2.26 | 11.3 |
| 25 | 0.03 | 0.34 | 1.58 | 0.39 | 1.8 | 1.95 | 65.0 |
|  | 0.05 | 0.34 | 1.88 | 0.42 | 1.78 | 2.36 | 47.2 |
|  | 0.075 | 0.52 | 1.59 | 0.33 | 1.74 | 2.31 | 30.8 |
|  | 0.1 | 0.62 | 1.26 | 0.67 | 1.58 | 2.28 | 22.8 |
|  | 0.2 | 0.84 | 1.08 | 0.82 | 1.53 | 2.26 | 11.3 |
| 35 | 0.03 | 0.32 | 1.57 | 0.4 | 1.73 | 1.88 | 62.6 |
|  | 0.05 | 0.35 | 1.54 | 0.42 | 1.78 | 2.25 | 45.0 |
|  | 0.075 | 0.58 | 1.44 | 0.61 | 1.47 | 2.20 | 29.3 |
|  | 0.1 | 0.64 | 1.53 | 0.69 | 1.5 | 2.17 | 21.7 |
|  | 0.2 | 0.82 | 1.53 | 0.82 | 1.55 | 2.12 | 10.6 |
| 45 | 0.03 | 0.31 | 1.57 | 0.4 | 1.71 | 1.75 | 58.3 |
|  | 0.05 | 0.34 | 1.58 | 0.44 | 1.85 | 2.06 | 41.2 |
|  | 0.075 | 0.58 | 1.36 | 0.62 | 1.72 | 2.01 | 26.8 |
|  | 0.1 | 0.68 | 1.64 | 0.68 | 1.66 | 1.98 | 19.8 |
|  | 0.2 | 0.81 | 1.56 | 0.82 | 1.54 | 1.94 | 9.7 |
| 55 | 0.03 | 0.29 | 1.61 | 0.43 | 1.82 | 1.61 | 53.8 |
|  | 0.05 | 0.34 | 1.55 | 0.47 | 1.99 | 1.89 | 37.8 |
|  | 0.075 | 0.59 | 1.46 | 0.63 | 1.58 | 1.83 | 24.4 |
|  | 0.1 | 0.68 | 1.4 | 0.7 | 1.45 | 1.81 | 18.1 |
|  | 0.2 | 0.82 | 1.58 | 0.82 | 1.5 | 1.78 | 8.9 |
| 65 | 0.03 | 0.29 | 1.64 | 0.45 | 1.94 | 1.45 | 48.4 |
|  | 0.05 | 0.29 | 1.64 | 0.45 | 1.94 | 1.65 | 32.9 |
|  | 0.075 | 0.6 | 1.54 | 0.69 | 1.51 | 1.61 | 21.5 |
|  | 0.1 | 0.65 | 1.64 | 0.72 | 1.53 | 1.59 | 15.9 |
|  | 0.2 | 0.84 | 1.55 | 0.84 | 1.11 | 1.59 | 7.9 |

***Table S3****. Discontinuous dilutions (D) with automatic harvesting at various culture system depths for optimised biomass production illustrated in figure 1a, panel iii. Areal and volumetric biomass production (AP, VP) have full units of g biomass-C m^-2^ d^-1^. harvest_point is the ratio biomass (day n):biomass (day n-1) used as a trigger to commence harvesting.*

| Latitude  (°) | Depth  (m) | D (d^-1^)  (Winter) | harvest_point  (Winter) | D (d^-1^)  (Summer) | harvest_point  (Summer) | AXP  (gCm^-2^d^-1^) | VXP  (gCm^-3^d^-1^) |
| --- | --- | --- | --- | --- | --- | --- | --- |
| 0 | 0.03 | 0.33 | 1.53 | 0.33 | 1.53 | 0.93 | 31.1 |
|  | 0.05 | 0.23 | 1.32 | 0.24 | 1.31 | 0.94 | 18.8 |
|  | 0.075 | 0.29 | 1.45 | 0.28 | 1.47 | 0.99 | 13.2 |
|  | 0.1 | 0.23 | 1.46 | 0.23 | 1.32 | 0.94 | 9.4 |
|  | 0.2 | 0.13 | 1.13 | 0.13 | 1.14 | 0.80 | 4.0 |
| 15 | 0.03 | 0.31 | 1.47 | 0.33 | 1.61 | 0.95 | 31.6 |
|  | 0.05 | 0.23 | 1.36 | 0.24 | 1.36 | 0.97 | 19.4 |
|  | 0.075 | 0.29 | 1.46 | 0.32 | 1.48 | 1.02 | 13.6 |
|  | 0.1 | 0.23 | 1.33 | 0.25 | 1.39 | 0.97 | 9.7 |
|  | 0.2 | 0.14 | 1.61 | 0.14 | 1.19 | 0.83 | 4.1 |
| 25 | 0.03 | 0.3 | 1.45 | 0.35 | 1.63 | 0.93 | 31.1 |
|  | 0.05 | 0.22 | 1.32 | 0.26 | 1.36 | 0.95 | 19.0 |
|  | 0.075 | 0.27 | 1.47 | 0.32 | 1.49 | 1.00 | 13.3 |
|  | 0.1 | 0.22 | 1.33 | 0.25 | 1.4 | 0.95 | 9.5 |
|  | 0.2 | 0.14 | 1.61 | 0.14 | 1.61 | 0.81 | 4.0 |
| 35 | 0.03 | 0.27 | 1.5 | 0.35 | 1.64 | 0.89 | 29.8 |
|  | 0.05 | 0.19 | 1.35 | 0.26 | 1.4 | 0.89 | 17.8 |
|  | 0.075 | 0.24 | 1.35 | 0.32 | 1.48 | 0.94 | 12.6 |
|  | 0.1 | 0.2 | 1.34 | 0.26 | 1.39 | 0.89 | 8.9 |
|  | 0.2 | 0.14 | 1.61 | 0.15 | 1.19 | 0.77 | 3.8 |
| 45 | 0.03 | 0.23 | 1.43 | 0.34 | 1.63 | 0.82 | 27.3 |
|  | 0.05 | 0.16 | 1.46 | 0.26 | 1.36 | 0.80 | 16.0 |
|  | 0.075 | 0.2 | 1.33 | 0.33 | 1.33 | 0.84 | 11.1 |
|  | 0.1 | 0.16 | 1.5 | 0.26 | 1.26 | 0.80 | 8.0 |
|  | 0.2 | 0.09 | 1.77 | 0.14 | 1.14 | 0.69 | 3.5 |
| 55 | 0.03 | 0.18 | 1.34 | 0.35 | 1.63 | 0.71 | 23.7 |
|  | 0.05 | 0.11 | 1.26 | 0.25 | 1.39 | 0.71 | 14.2 |
|  | 0.075 | 0.14 | 1.32 | 0.32 | 1.47 | 0.74 | 9.8 |
|  | 0.1 | 0.12 | 1.25 | 0.25 | 1.39 | 0.69 | 6.9 |
|  | 0.2 | 0.09 | 1.3 | 0.15 | 1.19 | 0.56 | 2.8 |
| 65 | 0.03 | 0.14 | 1.19 | 0.35 | 1.63 | 0.61 | 20.4 |
|  | 0.05 | 0.09 | 1.12 | 0.24 | 1.38 | 0.59 | 11.9 |
|  | 0.075 | 0.13 | 1.17 | 0.32 | 1.53 | 0.64 | 8.6 |
|  | 0.1 | 0.09 | 1.12 | 0.21 | 1.62 | 0.60 | 6.0 |
|  | 0.2 | 0.52 | 1.03 | 0.14 | 1.17 | 0.48 | 2.4 |

***Table S4****. Discontinuous dilutions (D) with automatic harvesting at various culture system depths for optimised biofuel production illustrated in figure 1b, panel iii. Areal and volumetric biofuels production (AXP, VXP) have full units of g biofuels-C m^-2^ d^-1^. harvest_point is the ratio biomass (day n):biomass (day n-1) used as a trigger to commence harvesting.*

|  |  | Biomass | | |  | Biofuel | | | | | | |
| --- | --- | --- | --- | --- | --- | --- | --- | --- | --- | --- | --- | --- |
|  |  |  |  |  |  | f/2 | | |  | f/4 | | |
| Latitude  (°) | Depth  (m) | D (d^-1^)  (Winter) | D (d^-1^)  (Summer) | AP  (gCm^-2^d^-1^) |  | D (d^-1^)  (Winter) | D (d^-1^)  (Summer) | AXP  (gCm^-2^d^-1^) |  | D (d^-1^)  (Winter) | D (d^-1^)  (Summer) | AXP  (gCm^-2^d^-1^) |
| Dilf = 1 day | | | | | | | | | | | | |
| 0 | 0.03 | 0.37 | 0.37 | 1.96 |  | 0.32 | 0.31 | 0.93 |  | 0.33 | 0.33 | 0.52 |
|  | 0.05 | 0.39 | 0.39 | 2.36 |  | 0.24 | 0.23 | 0.94 |  | 0.33 | 0.33 | 0.83 |
|  | 0.075 | 0.39 | 0.39 | 2.55 |  | 0.17 | 0.16 | 0.85 |  | 0.29 | 0.28 | 0.99 |
|  | 0.1 | 0.39 | 0.39 | 2.60 |  |  |  |  |  | 0.23 | 0.23 | 0.94 |
|  | 0.2 | 0.39 | 0.39 | 2.61 |  |  |  |  |  | 0.12 | 0.13 | 0.79 |
| 25 | 0.03 | 0.34 | 0.39 | 1.95 |  | 0.29 | 0.34 | 0.93 |  | 0.31 | 0.35 | 0.52 |
|  | 0.05 | 0.35 | 0.42 | 2.37 |  | 0.22 | 0.26 | 0.95 |  | 0.31 | 0.35 | 0.82 |
|  | 0.075 | 0.35 | 0.42 | 2.57 |  | 0.15 | 0.18 | 0.86 |  | 0.27 | 0.31 | 1.00 |
|  | 0.1 | 0.35 | 0.42 | 2.63 |  |  |  |  |  | 0.22 | 0.25 | 0.95 |
|  | 0.2 | 0.35 | 0.42 | 2.64 |  |  |  |  |  | 0.12 | 0.14 | 0.81 |
| 45 | 0.03 | 0.27 | 0.4 | 1.74 |  | 0.23 | 0.34 | 0.82 |  | 0.27 | 0.36 | 0.49 |
|  | 0.05 | 0.28 | 0.43 | 2.07 |  | 0.16 | 0.25 | 0.80 |  | 0.25 | 0.36 | 0.75 |
|  | 0.075 | 0.28 | 0.43 | 2.21 |  | 0.12 | 0.18 | 0.73 |  | 0.2 | 0.31 | 0.85 |
|  | 0.1 | 0.28 | 0.43 | 2.25 |  |  |  |  |  | 0.16 | 0.26 | 0.80 |
|  | 0.2 | 0.28 | 0.43 | 2.25 |  |  |  |  |  | 0.09 | 0.14 | 0.69 |
| 65 | 0.03 | 0.14 | 0.44 | 1.34 |  | 0.12 | 0.34 | 0.60 |  | 0.14 | 0.38 | 0.36 |
|  | 0.05 | 0.12 | 0.45 | 1.58 |  | 0.08 | 0.23 | 0.59 |  | 0.12 | 0.36 | 0.55 |
|  | 0.075 | 0.12 | 0.45 | 1.67 |  | 0.04 | 0.17 | 0.53 |  | 0.1 | 0.32 | 0.63 |
|  | 0.1 | 0.12 | 0.45 | 1.71 |  |  |  |  |  | 0.08 | 0.21 | 0.60 |
|  | 0.2 | 0.08 | 0.45 | 1.73 |  |  |  |  |  | 0.03 | 0.13 | 0.49 |
| Dilf = 2 days | | | | | | | | | | | | |
| 0 | 0.03 | 0.59 | 0.58 | 1.73 |  | 0.47 | 0.47 | 0.75 |  | 0.48 | 0.48 | 0.39 |
|  | 0.05 | 0.54 | 0.53 | 2.17 |  | 0.38 | 0.38 | 0.89 |  | 0.48 | 0.48 | 0.64 |
|  | 0.075 | 0.59 | 0.58 | 2.30 |  | 0.27 | 0.27 | 0.81 |  | 0.46 | 0.45 | 0.86 |
|  | 0.1 | 0.59 | 0.58 | 2.38 |  |  |  |  |  | 0.39 | 0.38 | 0.88 |
|  | 0.2 | 0.57 | 0.57 | 2.42 |  |  |  |  |  | 0.27 | 0.26 | 0.81 |
| 25 | 0.03 | 0.55 | 0.63 | 1.73 |  | 0.46 | 0.48 | 0.75 |  | 0.47 | 0.48 | 0.39 |
|  | 0.05 | 0.54 | 0.59 | 2.19 |  | 0.36 | 0.42 | 0.89 |  | 0.47 | 0.48 | 0.64 |
|  | 0.075 | 0.54 | 0.6 | 2.33 |  | 0.3 | 0.3 | 0.83 |  | 0.43 | 0.46 | 0.86 |
|  | 0.1 | 0.54 | 0.6 | 2.41 |  |  |  |  |  | 0.36 | 0.41 | 0.89 |
|  | 0.2 | 0.53 | 0.61 | 2.45 |  |  |  |  |  | 0.24 | 0.29 | 0.80 |
| 45 | 0.03 | 0.47 | 0.67 | 1.60 |  | 0.38 | 0.48 | 0.67 |  | 0.44 | 0.67 | 0.39 |
|  | 0.05 | 0.47 | 0.67 | 1.94 |  | 0.29 | 0.4 | 0.76 |  | 0.4 | 0.67 | 0.60 |
|  | 0.075 | 0.46 | 0.61 | 2.03 |  | 0.22 | 0.31 | 0.72 |  | 0.36 | 0.46 | 0.74 |
|  | 0.1 | 0.45 | 0.63 | 2.08 |  |  |  |  |  | 0.29 | 0.41 | 0.76 |
|  | 0.2 | 0.45 | 0.63 | 2.12 |  |  |  |  |  | 0.18 | 0.26 | 0.68 |
| 65 | 0.03 | 0.26 | 0.69 | 1.20 |  | 0.22 | 0.67 | 0.53 |  | 0.25 | 0.68 | 0.31 |
|  | 0.05 | 0.22 | 0.67 | 1.51 |  | 0.16 | 0.41 | 0.56 |  | 0.25 | 0.68 | 0.47 |
|  | 0.075 | 0.2 | 0.64 | 1.57 |  | 0.1 | 0.3 | 0.53 |  | 0.22 | 0.68 | 0.57 |
|  | 0.1 | 0.2 | 0.64 | 1.61 |  |  |  |  |  | 0.16 | 0.4 | 0.56 |
|  | 0.2 | 0.19 | 0.64 | 1.65 |  |  |  |  |  | 0.07 | 0.24 | 0.50 |
| Dilf = 4 days | | | | | | | | | | | | |
| 0 | 0.03 | 0.85 | 0.85 | 1.35 |  | 0.73 | 0.73 | 0.51 |  | 0.74 | 0.74 | 0.26 |
|  | 0.05 | 0.8 | 0.8 | 1.95 |  | 0.48 | 0.48 | 0.66 |  | 0.74 | 0.74 | 0.43 |
|  | 0.075 | 0.72 | 0.72 | 2.24 |  | 0.43 | 0.43 | 0.75 |  | 0.73 | 0.73 | 0.63 |
|  | 0.1 | 0.69 | 0.68 | 2.25 |  |  |  |  |  | 0.48 | 0.48 | 0.66 |
|  | 0.2 | 0.72 | 0.72 | 2.31 |  |  |  |  |  | 0.38 | 0.37 | 0.73 |
| 25 | 0.03 | 0.82 | 0.87 | 1.34 |  | 0.49 | 0.75 | 0.47 |  | 0.49 | 0.75 | 0.24 |
|  | 0.05 | 0.77 | 0.84 | 1.94 |  | 0.47 | 0.74 | 0.72 |  | 0.49 | 0.75 | 0.39 |
|  | 0.075 | 0.7 | 0.77 | 2.25 |  | 0.4 | 0.44 | 0.74 |  | 0.49 | 0.75 | 0.59 |
|  | 0.1 | 0.68 | 0.72 | 2.27 |  |  |  |  |  | 0.47 | 0.74 | 0.72 |
|  | 0.2 | 0.69 | 0.74 | 2.34 |  |  |  |  |  | 0.34 | 0.39 | 0.73 |
| 45 | 0.03 | 0.74 | 0.88 | 1.24 |  | 0.48 | 0.76 | 0.47 |  | 0.49 | 0.76 | 0.24 |
|  | 0.05 | 0.67 | 0.83 | 1.72 |  | 0.41 | 0.75 | 0.65 |  | 0.48 | 0.76 | 0.40 |
|  | 0.075 | 0.63 | 0.78 | 1.95 |  | 0.34 | 0.44 | 0.64 |  | 0.46 | 0.75 | 0.57 |
|  | 0.1 | 0.63 | 0.73 | 1.99 |  |  |  |  |  | 0.41 | 0.75 | 0.65 |
|  | 0.2 | 0.62 | 0.76 | 2.04 |  |  |  |  |  | 0.31 | 0.4 | 0.63 |
| 65 | 0.03 | 0.42 | 0.89 | 0.88 |  | 0.37 | 0.76 | 0.37 |  | 0.38 | 0.77 | 0.20 |
|  | 0.05 | 0.41 | 0.85 | 1.27 |  | 0.28 | 0.75 | 0.50 |  | 0.38 | 0.77 | 0.31 |
|  | 0.075 | 0.38 | 0.8 | 1.50 |  | 0.19 | 0.44 | 0.47 |  | 0.35 | 0.77 | 0.43 |
|  | 0.1 | 0.35 | 0.75 | 1.57 |  |  |  |  |  | 0.28 | 0.76 | 0.50 |
|  | 0.2 | 0.16 | 0.78 | 1.60 |  |  |  |  |  | 0.14 | 0.4 | 0.47 |

***Table S5****. Discontinuous dilutions (D) with prescribed harvest frequencies of dilf = 1, 2 and 4 days at various culture system depths for optimised biomass (see figure 2) and biofuel (see figure 3) production. Biomass production is supplied at f/2 nutrient concentrations (see Methods in the main text). Biofuel production is mainly supplied at f/4 levels but f/2 levels are compared for shallow depths where such concentrations can still lead to nutrient limitation.*
